# Molecular and serologic characterization of rotavirus from children with acute gastroenteritis in northern Iran, Gorgan

**DOI:** 10.1186/s12876-019-1025-x

**Published:** 2019-06-20

**Authors:** Nazanin Lorestani, Abdolvahhab Moradi, Ali Teimoori, Maha Masodi, Sayyad Khanizadeh, Mina Hassanpour, Naemeh Javid, Abdollah Ardebili, Alijan Tabarraei, Hadi Razavi Nikoo

**Affiliations:** 1 Laboratory Sciences Research Center, Golestan University of Medical Sciences, Gorgan, Iran; 2 Department of Microbiology, Faculty of Medicine, Golestan University of Medical Sciences, Gorgan, Iran; 3 Department of Microbiology, Faculty of Medicine, Hamadan University of Medical Sciences, Hamadan, Iran; 4 Hepatitis Research Center, Lorestan University of Medical Sciences, Khorramabad, Iran; 5 Department of Virology, Faculty of Medicine, Lorestan University of Medical Sciences, Khorramabad, Iran

**Keywords:** Rotavirus, Genotyping, Serotyping, Gastroenteritis, Iran

## Abstract

**Background:**

The pattern and distribution of human rotavirus genotypes in young children in developing countries play an important role in epidemiological studies, as well as providing a strategy for the development of future rotavirus vaccine.

**Methods:**

We evaluated stool samples from 349 children with acute gastroenteritis from Northern Iran (Gorgan city, Golestan province). Polyacrylamide Gel Electrophoresis (PAGE) and Latex Agglutination Test (LAT) were utilized to determine the prevalence of human rotavirus in fecal samples. Moreover semi-multiplex RT-PCR technique was carried out in order to determine the P and G genotypes of human rotavirus in rotavirus-positive samples.

**Results:**

A total of 46 rotavirus-positive samples were G and P genotyped. Whereas 28 (60.8%) fecal specimens contained only one rotavirus strain, 14 (30.4%) were mixed rotavirus infections and 4 (8.8%) was non-typeable. Overall, during the study, 57.82% of strains identified as genotype G1, G2 (18.70%), G3 (4.69%), G4 (3.13%), G8 (3.13%), G9 (6.26%) and non-typeable G (6.26%). From all these mentioned rotavirus strains, 46 were characterized as P [8] (97.80%) and P [4] (2.20%).Our analysis of the G and P genotyping of strains from all 46 rotavirus-infected children has revealed that 4/46(6.26%) of G type strains were non-typeable. The predominant single G/P combination was G1P [8] (57.82%), followed by, G2P [8] (16.98%), G2P [4] (1.72%), G3P [8] (4.69%), G4P [8] (3.13%) G8P [8] (3.13%), G9P [8] (6.26%) and four cases of non-typeable G (6.26%). Rotavirus was detected in 39 specimens (11.17%) by PAGE and in 38 specimens (10.88%) by LAT. Both tests were 100% specific; however, the LAT was 82.61% sensitive compared to the PAGE, which was 84.78% sensitive.

**Conclusions:**

The results suggest that to characterize rotavirus strains as well as design new effective vaccines for children with acute gastroenteritis, a large-scale study is needed in future.

## Background

Diarrhea disease is the eighth leading cause of annual death among all ages for more than1.6 million deaths and the fifth leading cause of death among under 5 years old children worldwide [1]. According to the World Health Organization (WHO), gastroenteritis affects generally 3 to 5 million children each year, of which nearly 12% are children younger than 5 years in 2015, responsible for nearly 446,000 deaths [1, 2]. The morbidity and mortality rate of gastroenteritis is increasing in developing countries. Acute gastroenteritis is one of the main public health problems caused by various well known pathogens, including: bacteria, protozoa and viral pathogens [3]. Recent studies have shown that enteric viruses, especially Rotavirus is the most important etiological agents for diarrhea mortality among children younger than 5 years with more than 128,000 deaths occur annually in the world [1, 4, 5]. The high percentage of rotavirus-related deaths (> 80%) is found in developing countries and it has been estimated that about 125 million episodes of diarrhea [6, 7]. These viruses are transmitted through fecal-oral route, which is known to be contagious from person to person which can happen by person to person contacts [7]. Rotaviruses, as member of Reoviridae family, are icosahedral, nonenveloped, and composed of three concentric layers of proteins with a core consisting of 11-segmented double-stranded RNA that code six structural (VP1-VP4, VP6 and VP7) and six nonstructural (NSP1-NSP6) proteins [8]. Serologically, human rotaviruses are divided into ten serogroups (A–J) and each group is based on genetic properties of major structural protein VP6 [9]. Group A rotaviruses are the major causes of acute diarrhea in infants and young children worldwide. Based on genetic diversity of two outer proteins, VP7 (glycosylated, G-type) and VP4 (protease sensitive, P-type), rotaviruses are classified in multiple serotypes, including 32 different G- and 47 different P genotypes [10]. There are several techniques for the detection of rotavirus in fecal samples. These tests including; virus cultivation, Electron Microscopy (EM), Enzyme-Linked Immunosorbent Assay (ELISA), Latex Agglutination Test (LAT), Polyacrylamide Gel Electrophoresis (PAGE) and Reverse Transcriptase Polymerase Chain Reaction (RT-PCR), which each of the methods have number of advantages and disadvantages [11]. In this regard, we designed this study to determine almost all known rotaviruses of the G and P genotypes in children with acute gastroenteritis in Gorgan, Iran. In this study, we used PAGE and latex agglutination tests for detection of rotavirus in fecal samples. Moreover, we compared the sensitivity and specificity of these tests in detection of rotavirus. To determine the P and G genotypes of rotavirus, semi-multiplex RT-PCR technique was carried on in this study. In addition, we reported genotype distribution and epidemiological data for rotavirus infections in samples collected during 1 year (2016–2017) from children with acute gastroenteritis, which admitted to Taleghani hospital, Gorgan, Iran.

## Methods

### Definition of diarrhea

The diarrhea is determined by the occurrence of three or more watery or loose stool daily lasting for at least 3 days [12].

### Ethic statement

This project was approved by the Ethic Committee of Golestan University of Medical Sciences (Ethic code: ir.goums.rec.1396.8), and it has been accomplished under supervision of an infection specialist they have been informed by doctors about this study. In addition, consent was obtained from their mothers.

### Fecal sample collection and preparation

From October 2016 to October 2017, a total of 349 diarrheic stool samples were collected from children (less than 5 years of old) were admitted at Taleghani hospital, Gorgan, Iran, with acute gastroenteritis symptoms including; with or without vomiting, fever, nausea, abdominal pain, and cramp. The fecal specimens were then transported on ice to the department of microbiology at Golestan university of Medical Sciences, Gorgan, Iran. The specimens were diluted with PBS 10%, and clarified by centrifugation. Clarified supernatants (1 ml) stored at 4-8 °C for short term storage or were stored at –70 °C for the longer term.

### Rotavirus antigen detection by latex agglutination test

Latex agglutination slide test, for screening and detection of VP6 group A rotavirus antigen, was used with the Plasmatec kit (LOT: 218028) following the manufacturer’s instructions for stool samples. All the material were brought to room temperature and mixed well before the test. This is a rapid slide test in which latex particles are coated with anti-rotavirus antibodies specific for group A rotavirus antigens present in a fecal supernatant. This test is interpreted able with the naked eye in few min [13].

### Polyacrylamide gel electrophoresis (Electropherotyping)

Polyacrylamide gel electrophoresis (PAGE) was performed with slab gels using standard Laemmli’s protocol [14]. For complete resolving of viral dsRNA, electrophoresis was performed at room temperature for 16 h at 20 mA and 70 V. After electrophoresis, gel was removed from between the glass plates, then fixed and stained simultaneously in a solution containing 5% ethanol, 1% nitric acid, and 0.1% AgNO_3_ for 5 min; thereafter, the solution was discarded. The gels were rinsed three times with distilled water for 10s, and then developed with a solution of 1.3% NaOH, 0.5% Na_2_CO_3_, and 0.4% HCOH (30%) for 1 to 2 min until the appearance of dark-stained bands on the yellow background. Development was stopped with a solution containing 5% ethanol and 1% nitric acid for 1 min, and the stopping solution was then discarded. Resulting migration patterns can then be visualized by staining the gel with silver nitrate [15, 16].

### Viral RNA extraction and reverse transcription

To extract viral RNA, a standard phenol–chloroform extraction method with *RNX-Plus* reagent (CinnaGen, Tehran, Iran) was used for stool suspension (prepared in PBS). A 400-μl from each fecal suspension was collected for total RNA extraction using RNX-Plus reagent (CinnaGen kit, Iran) according to the manufacturer’s protocol. RNA extracts were stored at − 70 °C for more analysis including; polyacrylamide gel electrophoresis (PAGE), reverse transcription polymerase chain reaction (RT-PCR). Following the RNA extraction, 6-μl extracted dsRNA was converted to cDNA, with a *High Capacity cDNA Reverse Transcription Kit* (Applied biosystems, USA). Before the reverse transcription reaction, the RNA samples were incubated at 97 °C for 10 min with random hexamer primer (1 μM final concentration). The initial reverse transcription reaction was carried out at 25 °C for 10 min, 37 °C for 120 min, followed by an inactivation step of 85 °C for 5 min.

### Semi-nested multiplex RT-PCR for G and P genotyping

Genotyping of positive samples were performed according to the WHO genotyping method. All of the rotavirus positive samples were tested by semi-nested RT-PCR with specific primers for regions of the genes encoding the VP7 (G-type) and VP4 (P-type). The appropriate forward and reverse primers for the identification of VP7 and VP4 genotypes of selected sample are presented in Table 1. First and second amplifications were performed as indicated in Table 2. The PCR products were electrophoresed using 2% agarose gel, and the G and P genotypes were determined by the sizes of the amplicons .

**Table 1:** VP7 and VP4 consensus and type-specific primers

Primer	Type	Sequence (5′→3′)	Position	PCR product (bp
*Universal primer*				
VP7 con1 Forward		ATGTATGGTATTGAATATACCAC	51–71	1014
VP7 con2 Reverse		GGTCACATCATACAATTCTAATC	1062–1040	
VP4 con1 Forward		TGGCTTCGCTCATTTATAGACA	2–23	877
VP4con1 Reverse		ATYTCHGACCAYTTATAHCC	878–859	
*G-typing (VP7)*				
Gcon Reverse		GGTCACATCATACAATTCT	1062–1044	
aBT1	G1	CAAGTACTCAAATCAATGATGG	314–335	749
aCT2	G2	CAATGATATTAACACATTTTCTGTG	411–435	652
Aust	G3	ACGAACTCAACACGAGARG	250–269	813
aDT4	G4	CGTTTCTGGTGAGGAGTTG	480–498	58
aAT8	G8	GTCACACCATTTGTAAATTCG	178–198	88
mG9	G9	CTTGATGTGACTAYAAATAC	757–776	305
*P-typing (VP4)*				
con3 Forward		TGGCTTCGCTCATTTATAGACA	11–32	
2 T-1	P [4]	CTATTGTTAGAGGTTAGAGTC	474–494	484
3 T-1	P [6]	TGTTGATTAGTTGGATTCAA	259–278	260
1 T-1	P [8]	TCTACTTGGATAACGTGC	339–356	346
4 T-1	P [9]	TGAGACATGCAATTGGAC	385–402	392

**Table 2:** G and P typing PCR programs

	Denaturation								Extension		
	Temperature	Time							Temperature		Time
			Denaturation		Annealing		Extension				
			Temp	Time	Temp	Time	Temp	Time	No. cycles		
G typing 1st round	94	5	94	1	42	2	72	1	35	72	7
2nd round	94	5	94	1	45	2	72	1	15	72	7
P typing * 1st round	94	5	94	1	52	1	72	1	35	72	7
2nd round	94	5	94	1	52	1	72	1	20	72	7

### Statistical analysis

Data were statistically analyzed by the software SPSS, version 19.0. Differences between the groups according to the variables were analyzed using the chi-square test. *P* values ≤0.05 were considered statistically significant.

## Results

### Characteristics of fecal specimens and demographic data

**Fig. 1:**
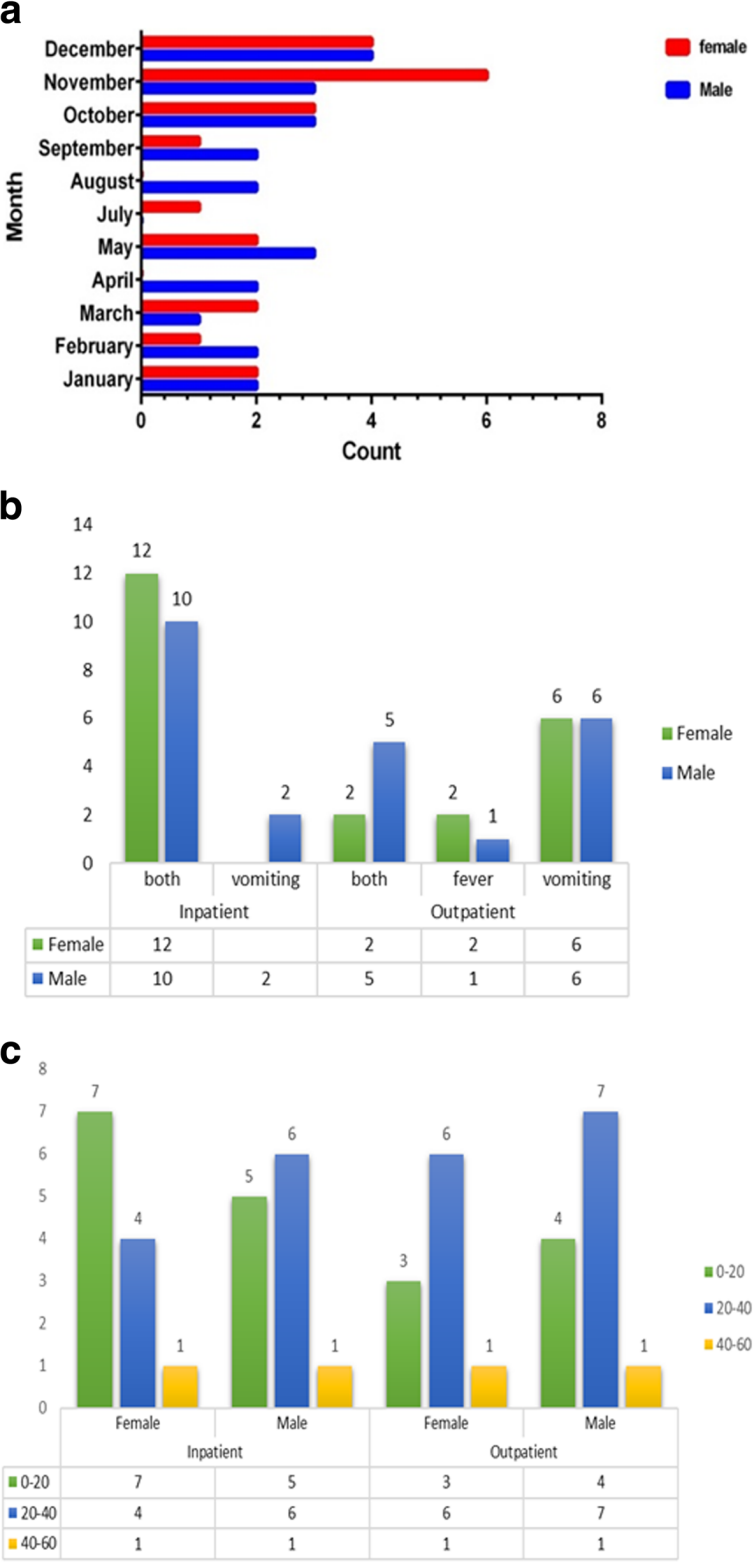
**a**. Monthly distribution of rotavirus detection in the Northern Iran, from 2016 to 2017. **b** Distribution of number of vomiting and fever in acute gastrointestinal cases tested positive for rotavirus from 2016 to 2017. **c** Age distribution of rotavirus detection rate in Northern Iran, from 2016 to 2017. Age ranges for each age group are indicated in color bars as follows: 0–20 months (Green bars), 20–40 months (Blue bars), 0–20 months (Yellow bars)

Three hundred forty-nine fecal samples were collected from 214 (61%) males and 135 (39%) females with acute gastroenteritis during October 2016 to October 2017. All the children with gastroenteritis were aged 0 to 60 months. Rotavirus was detected more in boys than (52.2%, 24/46) in girls (47.8%, 22/46). The age group analysis of rotavirus-positive cases revealed that the highest infection rate was among children between 20 and 40 months, with a mean age of 13.40 ± 11.01 months, while those within the age ranges of 0–2 and 48–59 months were the least affected, because no rotavirus infection was detected. The study of clinical pictures in positive cases showed that most children with infection had diarrhea (96.25%), vomiting (93.4%) and fever (69.5%).Rotavirus was detected throughout the year but relative frequency of rotavirus gastroenteritis was the highest in autumn. The seasonal distribution of rotavirus infection was as follow: 21.7% in spring, 6.5% in summer, 39.1% in autumn, and 32.6% in winter. A significant relationship was also found between rotavirus infection and seasonal distribution (*P* < 0.05) and the highest rate of detection of rotavirus gastroenteritis was found in November (19%) and the lowest in the months Jun and July, in which no infection was detected (*P* = 0.04) (Fig. 1A-C).

### Polyacrylamide gel electrophoresis analysis

**Fig. 2:**
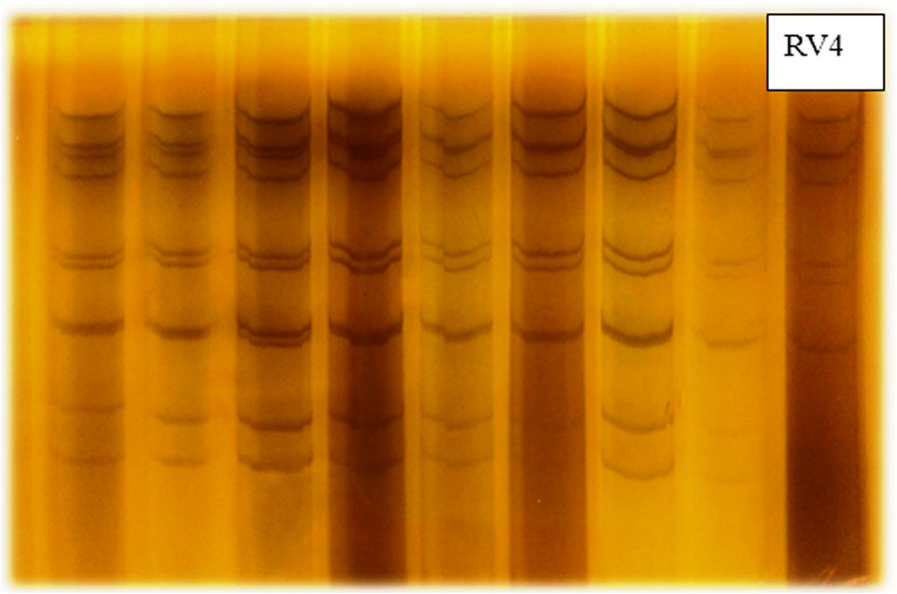
Electrophoretic migration pattern of RNA from rotavirus strains isolated. RV4 is used as the marker. The viral RNAs were analyzed by RV4 electrophoresis in a 12% polyacrylamide gel for 16 h and visualized by silver nitrate

In this study, initial screening for presence of group A human rotavirus genome in 345 fecal samples was performed by direct dsRNA extraction of samples with RNX-Plus reagent and RNA–PAGE analysis following the previously described procedure. Electrophoretic migration pattern of dsRNA genome of group A rotavirus (4:2:3:2), was detected in all positive (39/349) fecal samples collected from diarrheic children. The results obtained by followed by PAGE analysis of the extract are shown in Fig. 2.

### Serologic identification of rotavirus

**Fig. 3:**
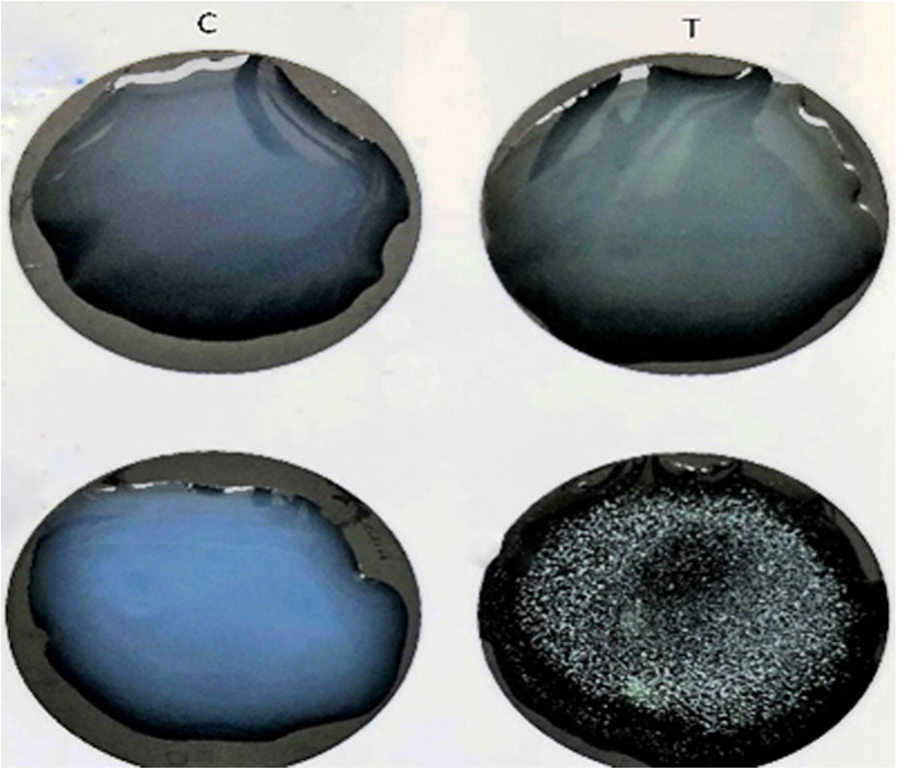
Latex agglutination test of fecal supernatant in patients with diarrhea. Agglutination of latex particles were occurred, when group A rotavirus antigens present in fecal sample and this agglutination interpreted with the naked eye in few min

In the present study, Latex slide agglutination test was carried out on 349 stool samples were collected from children aged < 5-years-old patients, suffering with diarrhea. In the procedure, a sample was considered positive for rotavirus when agglutination was observed within two minutes reaction, as indicated in Fig. 3. Latex slide agglutination test was positive in 38 of 349 (10.88%) stool specimens for the presence of group A rotavirus antigen.

### Identification of G and P genotypes in rotavirus-positive specimens

**Fig. 4:**
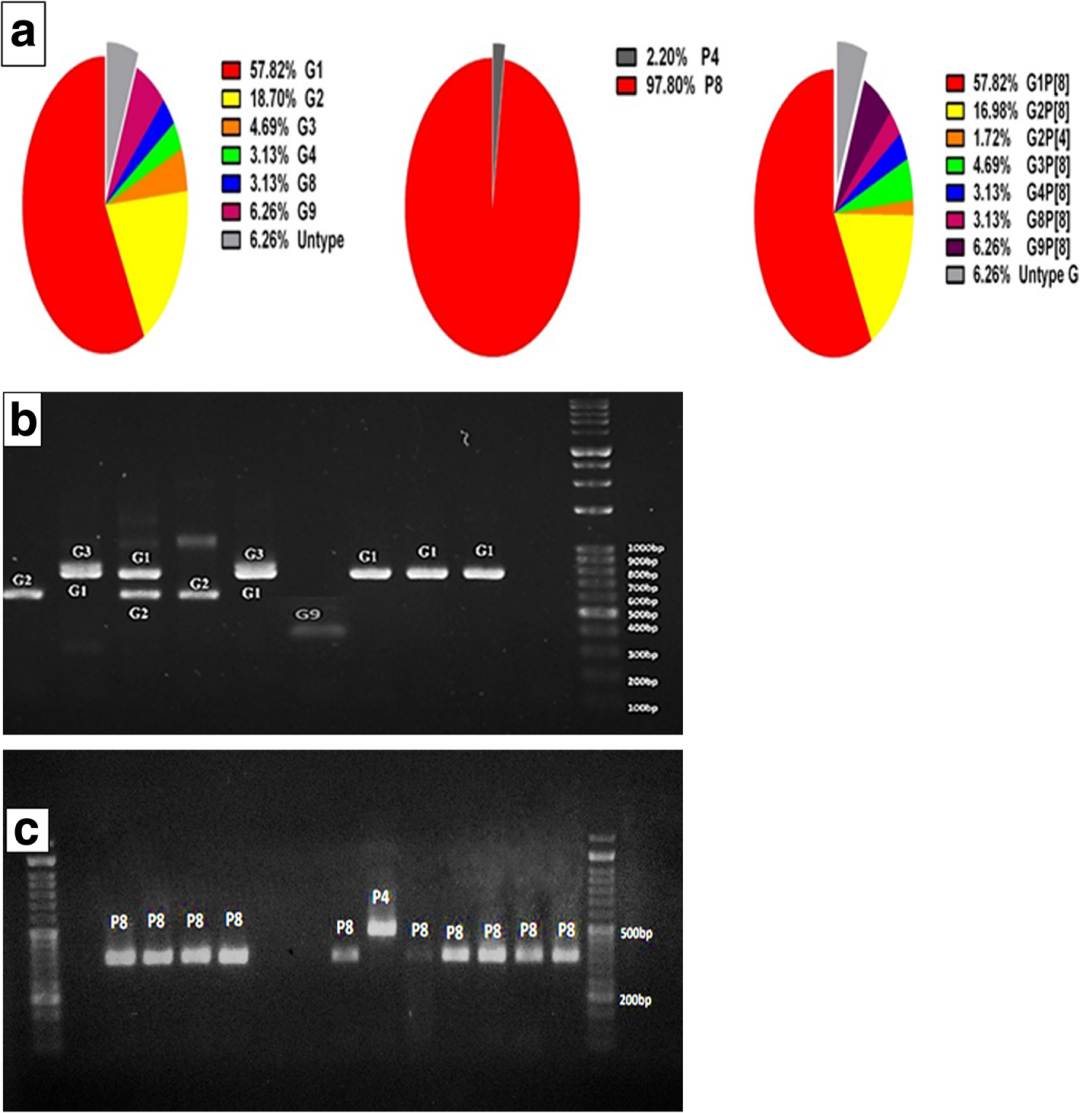
Distribution of G and P types found in 46 rotavirus-positive fecal samples in Northern Iran from 2016 to 2017. Figure 4 **a** Pie chart of G Genotypes, P Genotypes and combination Genotypes found in the fecal samples of acute gastrointestinal patients (denoted by their percentages). **b** Representative agarose gel electrophoresis of PCR products of G typing. **c** Representative agarose gel electrophoresis of PCR products of semi-multiplex P typing

According to RT-PCR analysis, a total of 46 samples were positive for rotavirus. Twenty-eight (60.8%) fecal specimens contained only one rotavirus strain and 14 (30.4%) were mixed rotavirus infections. Overall, during the study, 57.82% of strains identified were genotype G1, 18.70% G2, 4.69% G3, 3.13% G4, 3.13%G8, 6.26% G9 and 6.26% non-typeable G, Of these rotavirus strains, 46 were characterized as P [8] (97.80%) and p [4] (2.20%).Our analysis of the G and P genotypes of strains from all 46 rotavirus-infected children revealed: 4/46(6.26%) G type strains were found to be non-typeable. The predominant single G/P combination was G1P [8] (57.82%), followed by, G2P [8] (16.98%), G2P [4] (1.72%), G3P [8] (4.69%), G4P [8] (3.13%) G8P [8] (3.13%), G9P [8] (6.26%) and four cases of non-typeable G (6.26%). Rotavirus was detected in 39 specimens (11.17%) by PAGE, in 38 specimens (10.88%) by LAT. Both tests were 100% specific; however, the LAT was 82.61% sensitive compared to the PAGE, which was 84.78% sensitive. The distribution of human rotavirus G/P combinations is shown in Fig. 4a. Amplicon sizes of P and G genotypes are presented in Fig. 4b and c, respectively.

### Comparison of commercially latex slide agglutination test with PAGE analysis

Fecal samples from 349 children with diarrhea were tested for Rotavirus by the Rotatest latex slide agglutination test, the PAGE. Rotavirus was detected in 39 specimens (11.17%) by PAGE, in 38 specimens (10.88%) by LAT. The concordance of results among the two tests is shown in Table 3. Both tests were 100% specific; however, the LAT was 82.61% sensitive compared to the PAGE, which was 84.78% sensitive. These results indicate that the LAT kit used for rotavirus detection on feces performed properly, with a great simplicity and speed.

**Table 3:** Comparison of outcomes from two testing methods for human rotavirus in total fecal samples

Test	True positive	False positive	True negative	False negative	Sensitivity	Specificity	Positive predictive value	Negative predictive value
LAT	38	2	301	8	82.61%	99.34%	95%	97.41%
PAGE	39	0	303	7	84.78%	100%	100%	97.74%

## Discussion

Rotavirus is the most common cause of non-bacterial and severe gastroenteritis in young children in different regions of world, including developed and developing countries. This pathogen is responsible for more than 1.5 million hospitalizations and more than 128,000 deaths in children younger than 5 years of age worldwide [17]. The current study provides beneficial data in the context of epidemiology of rotavirus infections in children and infants with acute gastroenteritis, northern Iran, Gorgan.

The prevalence of rotavirus is dependent to several factors, including season of sampling, demographic (e.g. age, sex) and ethnic factors, and the specificity and sensitivity of the diagnostic methods [4, 18]. In a recent study, Monavari et al., reported a range of 6.4 to 79.3% of rotavirus infection, with the mean prevalence of 39.9% among the Iranian children [19]. According to the recent epidemiologic studies, increased worldwide prevalence of rotavirus among children aged less than 5 years in 1986–1999 was 20%, in 1990–2004 was 29%, and in 2001–2008 was 40% [20]. Similarly, up to 42.77% of rotavirus prevalence was reported in the Eastern Mediterranean region in 2010–2015 [21]. The rate of rotavirus in the present study was 13%. This widespread variability may be explained by above-mentioned variables.

Regarding this fact that rotavirus is considered as an etiologic agent of severe gastroenteritis in young children, screening and identification of patients in developing countries seems to be necessary. There are three categories of diagnostic methods for detection and typing of rotavirus from fecal specimens as follow: 1) Electron microscopy (EM) and nucleic acid detection by poly acrylamide gel (RNA-PAGE), 2) Antigen detection tests, including enzyme immunoassay (EIA), latex agglutination tests (LAT), and 3) nucleic acid amplification assay (RT-PCR) [11, 22]. EM is highly specific and sensitive for detection of rotavirus. However, it is a time-consuming and costly technique and requires a highly trained technician. In addition, EM cannot distinguish different groups of rotaviruses [11, 22]. Because of the large quantities of rotavirus presented in stool samples, RNA-PAGE can be used for detection and typing of human rotaviruses. Like EM method, this method is time-consuming and requires a trained technologist. However, this method is powered by several advantages, including good sensitivity, detection of small amounts of virus, lack of ambiguity in the results and distinguishing different rotaviruses [11, 22]. Serological tests, such as ELISA and LAT are the commonly methods used for rotavirus diagnosis based on antigen detection in stool material. The LAT is a simple, rapid, sensitive and specific for routine performance in laboratories [23, 24].

In our study, 11.17 and 10.88% of specimens were positive for group A human rotavirus by PAGE and LAT, respectively. LAT has been shown as a method with good sensitivity and specificity in previous studies. One study conducted by Al-Yousif et al, the sensitivity and specificity of LAT in compared with ELISA were obtained 100 and 96.3%, respectively [23]. In second study, Haikala et al, reported that LAT is more sensitive than EM and ELISA assays [25]. Similarly, the LAT assay in the current study showed enough sensitivity and specificity. According to the fact that this method is easy to perform in short time and does not need expensive equipment, the LAT could be recommended for routine performance in laboratories.

The results of P and G typing in this study revealed that genotype G1P [8] was dominant with the prevalence of 57.82%, followed by the genotypes G2P [8] (16.98%), G2P [4] (1.72%) G3P [8] (4.69%), G4P [8] (3/13%), G8P [8] (3.13%) and G9P [8] (6.26%) and non-typeable G (6.26%). The G1P [8], G2P [4], G3P [8], G4P [8], G9P [8], and G9P [6], have been reported as the most dominant genotypes in the USA and most European countries [21]. In a recent study, Azaran et al, reported the prevalence rate of 28.13% for genotype G9P [8] in patient with gasteroentrititis during 2015 to 2016 [26]. In contrast, G1P [8] was found as dominant genotype (57.82%) among the patient studied in the current study. In agreement with our study, previous studies conducted in Iran found similar findings [21, 27], Modaress et al, found G1P [8] as dominant genotype with a prevalence rate of 53.4% [28].

Here, it was found that there was no significant correlation between the rotavirus genotypes and clinical features, a figure that is inconsistent with by previous studies [13, 21, 29]. Rotavirus was shown in tropical climates throughout the year and also, its seasonal trends are less well defined. In this study, we found a significant correlation between the rotavirus and season, in agreement with other investigations [18]. Rotavirus-related gastroenteritis in our enrolled patient was occurred in autumn, with peaks in November and December.

Until recently, more than 60 countries have been performed vaccination of children against rotavirus and some of these countries demonstrated decreases of 22–50% in diarrhea mortality among children < 5 years of age [29]. One of the most important challenges for future rotavirus vaccine is absence of epidemiological studies and sufficient information about geographical distribution of different human rotavirus strains. Furthermore, it has demonstrated the importance and need for production of polyvalent rotavirus vaccine against circulating new rotavirus strains as suggested by WHO, which will help measures to control rotavirus epidemics in the future.

## Conclusion

This study emphasizes to conduct a large-scale study to characterize rotavirus strains and to determine their trend over time among Iranian children with acute gastroenteritis using valid and rapid laboratory technique. To design new effective vaccines for infant’s aged under 5-years-old, such studies appear to be necessary.

## Data Availability

The datasets used and/or analyzed during the current study are available from the corresponding author on reasonable request.

## Abbreviations

dsRNA: double stranded RNA
EIA: Enzyme Immunoassay
ELISA: Enzyme-Linked Immunosorbent Assay
EM: Electron microscopy
LAT: Latex Agglutination Tests
PAGE: Polyacrylamide Gel Electrophoresis
RT-PCR: Reverse Transcriptase-Polymerase Chain Reaction
RV: Rotavirus
WHO: World Health Organization
